# Glucotoxicity Induced Oxidative Stress and Inflammation* In Vivo* and* In Vitro* in* Psammomys obesus*: Involvement of Aqueous Extract of* Brassica rapa rapifera*

**DOI:** 10.1155/2016/3689208

**Published:** 2016-03-07

**Authors:** Sihem Berdja, Leila Smail, Boualem Saka, Samia Neggazi, El-mehdi Haffaf, Yasmina Benazzoug, Ghouti Kacimi, Lynda Boudarene, Souhila Aouichat Bouguerra

**Affiliations:** ^1^Laboratory of Physiology of Organisms, Team of Cellular and Molecular Physiopathology, Faculty of Biological Sciences, University of Technological Sciences Houari Boumediene, BP 32, EL Alia, 16011 Algiers, Algeria; ^2^Laboratory of Organic and Functionally Analysis, Faculty of Chemistry, USTHB, BP 32, El Alia, 16011 Algiers, Algeria; ^3^Laboratory of Nuclear Medicine of Central Hospital of Army, Ain Naadja, 16005 Algiers, Algeria; ^4^Team of Biochemistry and Extracellular Matrix Remodeling, Faculty of Biological Sciences, USTHB, BP 32, El Alia, 16011 Algiers, Algeria; ^5^Laboratory of Biochemistry of Central Hospital of Army, Ain Naadja, 16005 Algiers, Algeria

## Abstract

*Context*.* Brassica rapa* is considered as natural source of antioxidants and is used to treat diabetes.* Objective*. Our study carried the impact of glucotoxicity induced* in vivo* and* in vitro* in vascular smooth muscle cells (VSMCs) in* Psammomys* and the therapeutic effect of* Brassica rapa* (AEBr).* Materials and Methods*. We administered a hyperglucidic diet (30% sucrose) for 9 months and a treatment for 20 days with AEBr at 100 mg/kg. VSMCs were submitted to D-Glucose (0.6%) for 48 hours and treated with AEBr (2100 *μ*g/mL) for 24 hours. We measured, in blood metabolic parameters, the redox statues and inflammatory markers in adipose tissue. Histological study was effectuated in liver. In VSMCs, we measured markers of glucotoxicity (IRS1p Serine, AKT) inflammation (NO, MCP1, TNF*α*, and NF-*κ*B) and oxidative stress (oxidants and antioxydants markers). Cell viability and apoptosis were estimated by the morphological study.* Results*. AEBr corrects the metabolic parameters and inflammatory and oxidative markers in blood and homogenate tissue and reduces lipid droplets in liver. It induces, in VSMCs, a significant decrease of IRS1p serine, cyt c, NO, MCP1, TNF*α*, NF-*κ*B, protein, and lipid oxidation and increases cell viability, AKT, ERK1/2, catalase, and SOD activity.* Conclusion*.* Brassica* enhanced the antidiabetic, anti-inflammatory, and antioxidant defense leading to the protection of cardiovascular diseases.

## 1. Introduction

In the current century, diabetes mellitus (DM) is the most common health problem in the word. Nowadays, more than 387 million people suffer from DM and 592 million are expected to be affected by diabetes in 2035 [1]. Now, there is no doubt that DM and its related complications are associated with increased oxidative stress resulting from the imbalance in the production of free radicals, such as reactive oxygen species (ROS) and the system body's antioxidant defense [2]. The ROS have an important role in the etiology of diabetes and its complications [3]. There are many targets of oxidative damage in the diabetic vasculature, with modifications of protein, lipid, and nucleic acids, occurring in both endothelial and smooth muscle cells [4]. Pathogenic conditions include micro- (neuropathy, retinopathy, and nephropathy) and macrovascular complications and a consequent decrease in quality of life and increase in the rate of mortality [5]. The chronic hyperglycemia induces a modulation of the expression of many protein keys, involved in glucose toxicity [6]. It induces a dysfunction of the intracellular signal transduction in modulating the activity of kinase C protein, generating oxygen reactive species, activating ER-stress, generating advanced glycation end products, activating polyol pathway and hexosamines, and increasing release of proinflammatory cytokines as well as growth factors. All these alterations lead to endothelial dysfunction, which may be regarded as a sign of vascular disease being a key factor in the development of atherosclerosis [6–8]. Hence, inhibition of ROS generation due to glucotoxicity can be conducted to an important independent strategy to restoring the normal functioning of liver and controlling the progression of damage in organs. One of the major cellular responses to high glucose induces stress and hence ROS generation, in mitochondria, is apoptotic cell death [9].

A variety of natural products have been proposed as pharmacological treatment of type 2 diabetes and metabolic syndrome.* Brassica rapa* or “Turnip” species constitutes one source of food and can be considered as an important natural source of antioxidant. Moreover, in traditional medicine,* Brassica rapa* is used to treat a variety of diseases such as hepatitis, jaundice, furuncle, and sore throats [10]. Turnip contains numerous biologically active compounds, such as flavonoids (isorhamnetin, kaempferol, and quercetin glycosides), phenylpropanoid derivatives [10], indole alkaloids, and sterol glucosides (glucosinolates) [11]. Previous studies indicated that the alcoholic extracts of* Brassica rapa* have antidiabetic and antioxidant activities [12–14].

The present study aims at evaluating the impact of glucotoxicity induced* in vivo* and* in vitro* in VSMCs in* Psammomys obesus* (*Po*) and the action of aqueous extract of* Brassica rapa* (AEBr) by evaluating of metabolism disorder, the glucotoxicity signaling pathways, and the inflammatory and stress markers.

## 2. Materials and Methods

### 2.1. Preparation of Aqueous Extract of* Brassica rapa var. rapifera*

Fresh roots of* Brassica rapa *var.* rapifera* have been collected, in February 2012, from Algeria's markets. Voucher specimen (INA/P/No. 6) has been kept in the herbarium of Botany Department, National Institute of Agronomy (INA), Algiers, Algeria. The roots were separated from turnip tops; each one has been cut into small slices and dried in shade till complete drying. The plant material was pulverized into powdered form. The aqueous extract was prepared by decocting powdered roots (100 g) three times till complete exhaustion. Then, the collected aqueous extract was then lyophilized (Cryodos 80, −75°C, 5 m^3^/h) to obtain extract in yield of 0.15%. The extract was stored in sealed glass vials at ±4°C prior to be tested and analyzed.

### 2.2. Animals

Our study is conducted on an experimental model of Gerbillidae class (*Psammomys obesus*), a deserticolous rodent from the region of Beni-Abbes, south west of Algeria, in city of Bechar (30°7′ northern latitude and 2°10′ western longitude). In its natural environment, this gerbil eats halophilic Chenopodiaceae poor in calories (0.4 Kcal/g for* Salsola foetida*) and rich in water and mineral salts especially the sodium salt [15]. After familiarization time, animals are divided into two groups, the first one for control and the second one is experimental: a control group (*n* = 12 animals) with average body weight of 94.83 ± 2.99 g receiving 50 g of halophilic plants daily, which corresponds to 20–22 calories of energy intake per animal and a hyperglucidic diet group (HGD) (*n* = 12 animals) with average body weight of 86.16 ± 4.87 g receiving daily 50 g of halophilic plants with added 30% sucrose, corresponding to 80–82 calories of energy intake per animal (1 g of sucrose equals 4 calories) for 9 months. In the last 20 days of experimentation, the control and HGD group divided into two subgroups: (1) control, (2) control supplemented with aqueous extract of* Brassica rapa* var.* rapifera* (AEBr), (3) HGD, and (4) HGD treated with AEBr (HGD + AEBr). Thereafter, AEBr (100 mg/kg of body weight/day in NaCl 0.9%) were force-fed for 20 days for 2 and 4 groups. Simultaneously, groups 1 and 3 were force-fed with similar volume of NaCl 0.9%. Another control group (*n* = 5) is used in* in vitro* study of aortic vascular smooth muscle cells (VSMCs).

### 2.3. Methods 

#### 2.3.1. Chemical Study

*Total Phenolic Content*. Total phenolic contents of the extract were determined using Folin-Ciocalteu reagent according to the method of Singleton et al. [16], using gallic acid as a standard, and as modified by Djouahri et al. [17]. An aliquot (0.2 mL) of extract solution containing 1000 *μ*g of extract was mixed with 46 mL of distilled water and 1 mL of Folin-Ciocalteu reagent in a volumetric flask. After spending 3 min in the dark, 3 mL of sodium carbonate solution (7.5%) was added. Absorbance at 740 nm was measured in a spectrophotometer (Shimadzu 1800, Mulgrave, Victoria, Australia) after shaking and spending an additional 2 h in the dark. The total phenolic content was evaluated from a standard calibration curve of gallic acid, and results were expressed as microgram of gallic acid (GA) equivalents (E) per milligram of extract (*μ*g GAE/mg).

*Determination of Total Flavonoids*. The total flavonoids were determined according to the Dowd method described by Djouahri et al. [17]. Diluted solution of extract (4 mL) was mixed with 4 mL of aluminum trichloride solution (2% in methanol). After 15 min, the absorbance was measured at 415 nm. Quercetin (Q) was used as reference compound to produce the standard curve. The results are expressed as *μ*g QE/mg.

*Antioxidant Activity: Scavenging Effect on DPPH Radical*. The 2,2-diphenyl-1-picrylhydrazyl (DPPH) free radical scavenging assay was carried out as described by Brand-Williams et al. [18]. The aqueous extract was dissolved in methanol. Sample of 25 *μ*L of each concentration (100, 200, 400, 600, 800, and 1000 *μ*g/mL) was added to the DPPH methanol solution (60 *μ*M, 975 *μ*L). After 30 min of incubation at 25°C, the absorbance at 517 nm was measured by using UV spectrophotometer (Jasco, V-530). Ascorbic acid and *α*-tocopherol were used as compounds reference. The radical scavenging activity was then calculated from the equation: % of radical scavenging activity = [(Abs_control_ − Abs_sample_)/Abs_control_] × 100, where Abs_control_ is the absorption of the blank sample and Abs_sample_ is the absorbance of the tested extract.

#### 2.3.2. Biological Study

*Analytical Techniques*. The animals were bled from the retroorbital venous plexus; this technique eliminates using anesthetic agents affecting measurements of biochemical parameters. Blood, which is collected in dried tubes, was centrifuged at 3000 rpm for 10 min and the sera were stored at −20°C. Blood glucose, triglyceride, cholesterol, and protein were measured by enzymatic colorimetric method using a test kit of Biosystem. Blood insulin was determined by radioimmunoassay using CIS test kit (ORIS INDUS). Blood creatine phosphokinase (CPK) and lactic dehydrogenase (LDH) were determined by the automated CKL, 0–323. The evaluation of the redox status was performed in the sera and erythrocytes by assaying the thiobarbituric acid reactive substances (TBARs) and catalase.

*Organs Harvesting*. At the end of the experiment, animals were sacrificed after anesthesia by intraperitoneal injection of urethane at 25%, at 0.4 mL/100 g body weight. Some aortas were harvested under sterile conditions for cell culture, the adipose tissue was frozen in liquid nitrogen for evaluation of inflammatory markers, and the liver was fixed in Bouin's solution for histology study.

*Histology of the Liver*. After fixation in Bouin's aqueous, the specimens of heart and liver were dehydrated, embedded in paraffin, and cut at 5 *μ*m. The sections were stained with Masson's trichrome [19].

*Aortic Smooth Muscle Cell Culture*. Cell culture technique was used according to Bourdillon et al. [20] and Aouichat Bouguerra et al. [21]. Explants were obtained from thoracic aorta of control* Psammomys obesus*; they were prepared after removing adventitia by collagenase action at 0.1% (type IA; Sigma, USA) and incubated in Dulbecco's modified Eagle's medium (DMEM) (Gibco, USA), supplemented with 20% fetal calf serum (Sigma, USA), 1% antibiotics (streptomycin 50 mg/mL, penicillin 50 UI/mL, Sigma), and 1.2% glutamine (Sigma). The explants were maintained at 37°C under air-CO_2_ (95%–5%) atmosphere until they reached confluence. Then, VSMCs were trypsinized (0.1% of trypsin; Gibco, USA) and subcultured. In this experiment, the VSMCs were used in the sixth passage.

*Glucose Effects and Treatment with* AEBr* on Smooth Muscle Cells in Culture*. At confluence, the VSMCs were suspended after trypsinisation in multiwells at 800 000 cells/Wells and were subdivided into groups: Group1: VSMCs are incubated in cultured medium (DMEM) supplemented with fetal calf serum (FCS), 1% antibiotics, and 1.2% L-glutamine for 48 h. After an incubation of 48 h, the VSMCs were subdivided into 2 subgroups: (1) control incubated in DMEM 10% FCS for 24 h; (2) control incubated in DMEM 10% FCS and treated with *Brassica rapa* var.* rapifera* at IC_50_ (2100 *μ*g/mL). Group 2: VSMCs are incubated in cultured medium (DMEM) supplemented with fetal calf serum (FCS), 1% antibiotics, and 1.2% L. Glutamine for 48 h was enriched with 0.6% of D-Glucose for 48 h. After 48 h of incubation, the group was subdivided into two subgroups: the first incubated in DMEM 10% in presence of D-Glucose for 24 h; the second incubated in DMEM 10% in presence of D-Glucose treated with AEBr at IC_50_ (2100 *μ*g/mL) for 24 h. At the end, the cells were incubated in DMEM 1.2% glutamine and 1% antibiotic without FCS for different study.

*Proliferation Study*. The VSMCs controls submitted to D-Glucose in presence or absence of* Brassica* were trypsinized (0.1% of trypsin, Gibco, USA) and the proliferation rate was performed on 100 *μ*L cell suspension in presence of trypan blue by counting on Mallassez Cell.

*Morphological Study*. In the term of experimentation, the mediums in the wells were eliminated and the cells were washed with a phosphate buffered saline (PBS 1x) (Gibco) and fixed in the aqueous Bouin and colored with May Grunwald-Giemsa (MGG) (Fluka; V/V). The observation was done with an inverted microscope (Zeiss).

#### 2.3.3. Measurement of Antioxidant Activity

*Determination of Catalase Activity*. The enzymatic activity of catalase was determined by the method of Claiborne [22]. The principle is based on the disappearance of H_2_O_2_ in the presence of the enzyme source at 25°C. Catalase was measured in sera, erythrocytes, and intracellular compartment of all VSMCs groups. For this, a mixture consists of 500 *μ*L phosphate buffer (KH_2_PO_4_, pH 7.4 and 0.1 M) and 487,5 *μ*L freshly prepared H_2_O_2_ (0.091 M) and 12.5 *μ*L samples. Absorbance is estimated at 560 nm in two times *t*_0_ and after two minutes. Erythrocytes, organs, and cells were lysed, before all assays, in lysis buffer.

*Determination of Superoxyde Dismutase Activity*. The superoxyde dismutase (SOD) activity was measured by inhibiting it through nitroblue tetrazolium (NBT) reduction [23]. The reaction mixture contained 50 mM K-phosphate buffer (pH 7.8), 13 mM methionine, 75 *μ*M NBT, 0.1 *μ*M EDTA, 4 *μ*M riboflavin, and required amount of enzyme extract. The reaction was started by adding riboflavin and placing the tubes under two 15 W fluorescent lamps for 15 min. A complete reaction mixture without enzyme, giving the maximal color, served as a control. A nonirradiated complete reaction mixture served as a blank. One unit of SOD activity was defined as the enzyme's amount which is required to cause 50% inhibition of NBT's reduction as monitored at 560 nm, which was measured according to the method of Giannopolitis and Ries [24].

*Determination of Thiobarbituric Acid Reactive Substances (TBARs)*. After reaction with thiobarbituric acid (TBA) (Sigma) [25], the TBARs were measured in sera, erythrocytes, and intracellular compartment of all VSMCs groups. The samples were centrifuged at 10 000 g for 20 min at 4°C in buffered (Na_2_HPO_4_/NaH_2_PO_4_) 0.2 M, pH 6.5. The MDA contained in the supernatant in presence of 10% trichloroacetic acid reacted with TBA and caused the formation of a red complex estimated at 532 nm.

*Determination of Nitrogen Monoxide (NO)*. NO formation is typically and indirectly assessed by determining the concentrations of nitrites and nitrates, which are products of NO's oxidative degradation. The intracellular compartments of all VSMCs groups were deproteinized by centrifugation at 10 000 g for 10 minutes at −20°C. The determination of nitrite and nitrate is produced directly from the obtained supernatant. The Griess reaction only allows the measurement of nitrite. Nitrates should be reduced to nitrite before quantifying. The concentration thus measured represents the sum of nitrites and nitrates. The conversion of nitrate into nitrite is based on a reduction's reaction by cadmium and regeneration by using a solution of CuSO_4_ in 5 mM glycine-NaOH buffer, in contact for 5 minutes. The nitrite bearing in all samples which are deproteinized and regenerated is quantified after addition of Griess reagent [0.1% N-(1 naphthyl) ethylenediamine dihydrochloride, 1% sulfanilamide, and 5% phosphoric acid]. OD reading is made at 543 nm [26].

*Assay of Protein Carbonyl Levels*. Protein carbonyls (PC) were measured in the intracellular compartments of all VSMCs groups according to the procedure described by Reznick and Packer [27] using dinitrophenylhydrazine (DNPH) reagent and spectrophotometric method. PC groups react with 2,4-dinitrophenylhydrazine (DNPH) to generate chromophoric dinitrophenylhydrazones. Protein was precipitated with trichloroacetic acid of 20% (w/v). The precipitates were dissolved in guanidine-HCl 6 M solution and the absorbance was measured at 370 nm. The results were expressed as a nanomoles of carbonyl groups per milligram of protein using a molar extinction coefficient of 22 000 M^−1^ cm^−1^.

*Assay of Plasma Advanced Protein Oxidation Products*. Spectrophotometric determination of advanced protein oxidation products (AOPP) levels was performed in the intracellular compartments of all VSMCs groups by modifying Witko-Sarsat's method [28]. Samples were prepared according to the following way: 200 *μ*L samples and then 100 *μ*L of 1.16 M potassium iodide added to each tube, followed by 200 *μ*L acetic acid two minutes later. The absorbance of the reaction mixture was immediately estimated at 340 nm against a blank containing 200 *μ*L of phosphate buffer saline, 100 *μ*L of potassium iodide, and 200 *μ*L of acetic acid. AOPP concentrations are expressed as micromoles/L of chloramine-T equivalents [29].

*Measurement of Inflammation Markers and Glucotoxicity Signaling Voice*. The assessment was determined by immunoenzymatic assay. Invitrogen ELISA Kits were used for measuring the levels of different markers in the intracellular compartments of all VSMCs groups and organs. IRS 1 [p S312], MCP1, Rt TNF-*α*, NF-kB p65, ERK1 / 2, AKT [S p 473], and cytochrome C were performed on all groups cells. MCP1, Rt TNF-*α*, and NF-kB p65 were performed in adipose tissue of all groups. The estimation is made by Elisa reader at 450 nm (BioTek Instruments).

*Statistical Analysis*. Data were analyzed with ANOVA using STATISTICA version 6 and completed with HSD Tukey test. The results were expressed as the mean ± standard deviation. The differences at level ^*∗*^ *p* < 0.05 were considered to be statistically significant.

## 3. Results

### 3.1. Chemical Study

Total phenolics and flavonoids contents are as follows. The aqueous extract showed a weak amount of phenolic and flavonoid compounds with values of 9.41 ± 0.18 *μ*g GAE/mg and 1.01 ± 0.09 *μ*g QE/mg, respectively.

*Antioxidant Activity*. The principle antioxidant activity is based on the electrons' availability for neutralizing any free radicals. In this study, the antioxidant activity was evaluated by using scavenging DPPH free radicals assay. The results of the aqueous extract and the positive controls butylhydroxyanisole (BHA), butylhydroxytoluene (BHT).

The investigated aqueous extract of roots* B. rapa* var.* rapifera* showed a weak antiradical activity with IC_50_ value of 2100 ± 13 *μ*g/mL, which was extremely lower than the high antioxidant effect of BHA and BHT (IC_50_ = 21.28 ± 0.12 *μ*g/mL and 12.76 ± 0.08 *μ*g/mL, resp.) (Table 1).

### 3.2. *In Vivo* Study

In our experiment, we noted a significative increase of the glycemia, triglyceridemia, and cholesterolemia as well as a decrease of the insulinemia in animals submitted to hyperglucidic diet compared to corresponding controls signs of diabetes mellitus' installation, accompanied with an increase of atherogenic markers such as LDL-cholesterol, CPK, and LDH and with a decrease of cardioprotective lipid HDL-cholesterol. We noted also an increase in creatinemia levels sign of kidney function's perturbation (Tables 2(a) and 2(b)). The treatment with* Brassica* corrected the metabolic disorders by reducing glycemia, triglyceridemia, cholesterolemia, and creatinemia levels and increased the levels of insulin compared to animals submitted to hyperglucidic diet (HGD) (Tables 2(a) and 2(b)), protected the animals vascular complication by decreasing in atherogenic markers, and increased the cardioprotective lipid (Table 2(a)). The evaluation of redox statues in blood (sera and erythrocytes) showed a significant decrease in the activity of catalase with an increase in the rate of TBARs in HGD group (Table 3). The treatment with* Brassica* increases significantly the activity of catalase and decreases the rate of TBARs (Table 3).

The estimation of inflammatory states in adipose tissue by rates of MCP1, TNF*α*, and NF-*κ*B showed significantly an increase in HGD group compared to controls (Table 4). The treatment with* Brassica* reduced significantly the rate of these markers (Table 4).

However, the treatment of control sand rats with* Brassica* has no effect in the variation of glycemia, triglyceridemia, cholesterolemia, and insulinemia and HDL-cholesterol, LDL-cholesterol, CPK, LDH, and creatine levels compared to controls (Tables 2(a) and 2(b)); we also did not note variation in the balance of redox states and inflammation (Tables 3 and 4).

**Figure 1:**
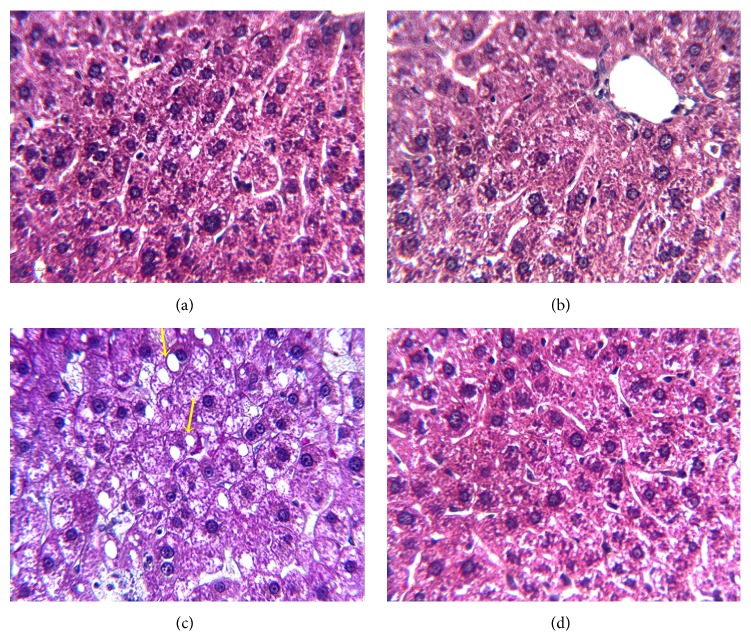
Liver histology after Masson's trichrome staining of liver sections from* Psammomys obesus* from each group: (a) control, (b) control + aqueous extract of* Brassica rapa* (AEBr), (c) hyperglucidic diet (HGD) (30% sucrose during 9th months), and (d) HGD + AEBr at 100 mg/kg for 20 consecutive days. Yellow arrow: hepatic lipid droplets.

The HGD group exhibited a higher hepatic lipid droplets compared to controls. The supplementation of* Brassica* lowered or made these lipid droplets disappear (Figure 1).

### 3.3. *In Vitro* Study

#### 3.3.1. Cells Viability

**Figure 2:**
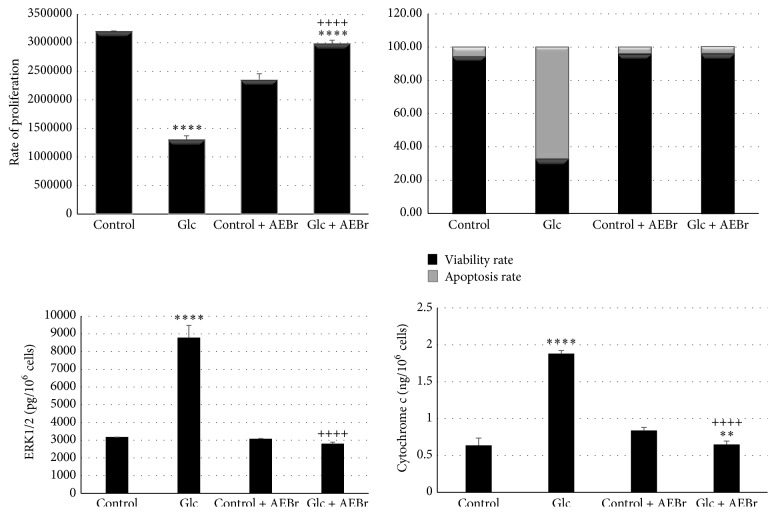
Estimation of the viability, the apoptosis, the rate of proliferation, the cytochrome c, and ERK1/2 of vascular smooth muscle cells (VSMCs) of* Psammomys obesus* submitted to glucotoxicity (Glc 0.6% for 48 hours). Effect of aqueous extract of* Brassica rapa* (AEBr) at 2100 *μ*g/mL for 24 hours. Data are expressed as mean ± standard deviation (S.D); (*n* = 6).* Po: Psammomys obesus;* Glc: D-Glucose. *p* > 0.5 denotes a statistical difference; *p* < 0.5 statistically different. *p* > 0.5 (*Po* control + AEBr versus* Po* control)*;* ^*∗∗*^ *p* < 0.01 (*Po* VSMCs Glc + AEBr versus* Po* control + AEBr), ^*∗∗∗∗*^ *p* < 0.0001 (*Po* VSMCs Glc + AEBr versus* Po* control + AEBr), ^*∗∗∗∗*^ *p* < 0.0001 (*Po* VSMCs GLC versus* Po* VSMCs control) and ^++++^ *p* < 0.0001 (*Po* VSMCs Glc + AEBr versus* Po* VSMCs Glc).

Our results showed a significant decrease in the rate of proliferation of VSMCs submitted to glucotoxicity (−59%) compared to control, accompanied with a significant decrease in the rate cell viability's rate and an increase in the cell death rate compared to control (Figure 2). The treatment with AEBr induced an increase of cell proliferation in cells in presence of Glc + AEBr compared to cells submitted to glucotoxicity (+129%) (Figure 2) and compared to control + AEBr (+26%).

We also noted a significant increase in the ERK1/2 (+176%) and the cytochrome c (+198%) levels in the cells submitted to glucotoxicity compared to control; the treatment with* Brassica* reduced significantly the levels. Treatment with AEBr reduced the rate of ERK1/2 at the same level of the controls. On the rate of cytochrome c, we noted a significant decrease compared to control in the presence of* Brassica* (Figure 2).

**Figure 3:**
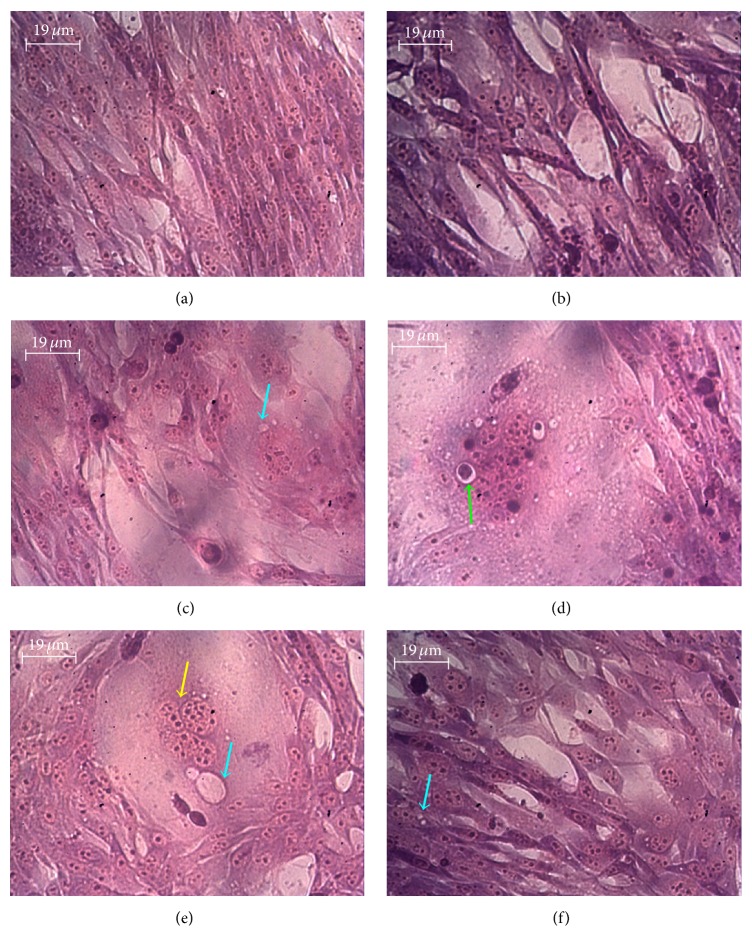
*Psammomys obesus* aortic smooth muscle cells (VSMCs), exposed at 0.6% of D-Glucose for 48 hours and treated with aqueous extract of* Brassica rapa* var.* rapifera* at 2100 *μ*g/mL for 24 hours. (a) Control, (b) control treated with AEBr, (c, d, e) VSMCs group exposure at high dose of D-Glucose, and (f) VSMCs exposure to glucotoxicity and treated with AEBr. The cells were fixed in aqueous Bouin and stained with May Grunwald-Giemsa (MGG); the observation was done with an inverted microscope (Zeiss) (GX400). Blue arrow: cytosolic vacuolization = oncosis, yellow arrow: fragmented nuclei fragmentation, and green arrow: apoptotic crop.

The VSMCs submitted to glucotoxicity showed morphological and functional alterations marked by cells' hypertrophy, cytosolic vacuolization due to oncosis, chromatin's hypercondensation, and fragmented nuclei indicating apoptosis alterations after MGG staining (Figure 3). Treatment with* Brassica* corrects slightly the morphological alterations marked by reduction in oncosis, hypertrophy, and the fluorescence (Figure 3).

#### 3.3.2. Glucotoxicity and Inflammatory Markers

**Figure 4:**
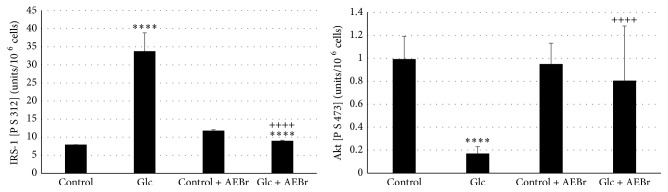
Evaluation of glucotoxicity signaling pathways (IRS1 P S 312 and AKT) in vascular smooth muscle's cells (VSMCs) of* Psammomys obesus* submitted to glucotoxicity (Glc 0.6% for 48 hours) and treated with aqueous extract of* Brassica rapa* (AEBr) at 2100 *μ*g/mL for 24 hours. Data are expressed as mean ± standard deviation (S.D)*;* (*n* = 6)*. Po: Psammomys obesus;* Glc: D-Glucose. *p* > 0.5 denotes a statistical difference; *p* < 0.5 statistically different. *p* > 0.5 (*Po* VSMCs control + AEBr versus* Po* VSMCs control)*;* ^*∗∗∗∗*^ *p* < 0.0001 (*Po* VSMCs Glc versus* Po* VSMCs control)*;* ^*∗∗∗∗*^ *p* < 0.001 (*Po* VSMCs Glc + AEBr versus* Po* control + AEBr)*;* ^++++^ *p* < 0.0001 (*Po* VSMCs Glc + AEBr versus* Po* VSMCs Glc).

The evaluation of the rates of glucotoxicity signaling pathways IRS1 Ps 312 and AKT showed a large increase of IRS1 and a decrease in AKT in VSMCs Glc group compared to control; the treatment with* Brassica* reduced significantly the levels of ERK1/2 and increased the levels of AKT to the same values of control in presence or absence of AEBr (Figure 4). Besides, the estimation of inflammatory markers in VSMCs Glc group showed an increase in the NO, MCP1, TNF*α*, and NF-*κ*B levels (Table 5). Treatment with AEBr reduced significantly the rate of these markers compared to controls in presence or absence of AEBr (Table 5). Little variation was observed between the two controls (Table 5).

#### 3.3.3. Redox Status

The evaluation of antioxidant activity by measurement of catalase and SOD activity in VSMCs submitted to glucotoxicity showed a decrease of catalase and SOD activity, compared to control. The treatment of VSMCs with AEBr enhances significantly the activity of catalase and SOD in Glc + AEBr group compared to Glc group and control + AEBr group. We noted a little variation in the SOD and catalase activities between controls which contained or did not contain* Brassica* (Table 6).

The assessment of stress markers such as AOPP, protein carbonyl in VSMCs Glc group, showed a very significant increase in the cellular compartment compared to control. The levels of TBARs evaluated in the intra- and extracompartment showed a significant increase notably in the extracompartment (Table 6). The treatment with* Brassica* reduced the levels of oxidative stress markers in VSMCs submitted to glucotoxicity compared to control groups in presence or absence of AEBr (Table 6).

## 4. Discussion

We analyzed, in the present study, the effect of glucotoxicity induced,* in vivo*, by high glucidic diet and,* in vitro,* by high concentration of glucose in VSMCs as well as evaluating the effect of aqueous extract of* Brassica rapa* in* Psammomys obesus*.

### 4.1. Chemical Study

Total phenolics and flavonoids contents were determined according to their importance as antioxidant compounds. In fact, there is a relationship between the antioxidant ability and the total phenol contents. Phenolic antioxidants are products of secondary metabolism in plants. The antioxidant activity is mainly due to their redox properties and chemical structure, playing an important role in chelating transitional metals, inhibiting lipoxygenase, and scavenging free radicals [30]. The principle antioxidant activity is based on electrons' availability to neutralize any free radicals. The antioxidant activity, in this study, was evaluated by determining IC_50_, using scavenging DPPH free radicals. The value of IC_50_ was used in the* in vitro* study. This activity could be explained by the presence of the phenolics compounds related to different mechanisms, such as free radical scavenging, hydrogen donation, singlet oxygen quenching, and metal ion-chelation, and acting as substrates for radicals such as superoxide and hydroxyl [31].

### 4.2. Metabolism Disorder

The long-term administration (9 months) of a high-carbohydrate diet caused both plasma and tissue disorders in* Psammomys,* by confirming its high sensitivity to caloric overload [32]. Our results confirm the work of Aouichat Bouguerra et al. [21] concerning* Psammomys* submitted to a high-carbohydrates diet, those of Shafrir [33] and Berdja et al. [19] in* Psammomys.*

We found, in our study, a hyperglycemia associated with hypertriglyceridemia, hypercholesterolemia, and hyperinsulinemia, followed by hypoinsulinemia at the end of experimentation. According to Stentz and Kitabchi [34] hyperglycemia is related to alterations in biochemical pathways which are responsible for micro- and macrovascular injuries related to endothelial dysfunction. Therefore, according to Marquie et al. [32] and Shafrir [33], the progression of diabetes in* Psammomys,* subjected to high-calorie diet, increased from hyperinsulinemia to hyperinsulinemia associated with hyperglycemia and then to an insulin deficiency due to apoptosis and necrosis of *β* cells.

Our results show the occurrence of dyslipidemia marked by an increase of trigliceridemia, cholesterolemia, and rate of LDL-cholesterol and decrease of HDL-cholesterol accompanied with hepatic lipid droplets. According to Navab et al. [35], HDL decreases atherosclerosis by protecting LDL against oxidation. However, chronic hyperglycemia can induce glycation of HDL and impair their protective functions which enhance the atherosclerotic process [36].

The administration of aqueous extract of* Brassica* improves the metabolic disorder. AEBr induces an antihyperglycemic effect by diminution of glucose levels, an augmentation of insulin, and corrects the dyslipidemia associated with an increase in the high density lipoprotein cholesterol. Our results showed a decrease of triglyceridemia and cholesterolemia and lowered hepatic lipid droplets after administration of* Brassica rapa* in diabetic rats; these results are consistent with many studies, which showed the antihyperglycemic effect of* Brassica* in diabetic rats [13, 37]. The antihyperglycemic effect was observed through administration of* Brassica* suggests whose extract may potentiate pancreatic secretion of insulin, increase the glucose uptake, or inhibit glucose absorption in gut [38]. AEBr improved dyslipidemia most probably due to presence of the flavonoid quercetin which is known to reduce hepatic fat accumulation and the presence of glucosinolates which are known to reduce triglycerides level [39].

In HGD groups, we show an elevation in serum creatinine concentration, indicating impairment in kidney function and increase of CPK and LDH levels indicator cardiac complications. In a state of necrosis, SMCs release enzymes and proteins, in particular creatine phosphokinase, into the blood stream. During a cardiac episode, the increase of CPK is systematic [40].

The administration of AEBr to HGD group corrects the markers' rate of nephropathy and cardiovascular complications in relation to glucotoxicity by reducing of creatinine, CPK, and LDH levels. According, respectively, to Kataya and Hamza [41] and Daryoush et al. [13],* Brassica* normalized the level of creatinine and LDH in diabetic rats. Brassicaceae contains anthocyanin pigments that are described as free radical scavenging and antioxidant agents.

### 4.3. Glucotoxicity and Inflammatory Status

The current study shows that glucotoxicity increases the level of IRS1-p-serine and impairs the activity of AKT in VSMCs. Hyperglycemia leads to the activation of multiple serine kinase cascades. There are a number of potential targets of these kinases in the insulin-signaling pathway, including the insulin receptor (IR) and the insulin receptor substrate (IRS) family of proteins. Increased phosphorylation of the IRS on serine sites, instead of tyrosine, indicates an impaired insulin action. In fact, the serine IRS1 phosphorylation is less able to associate with the downstream target molecules, especially phosphatidylinositol 3-kinase/AKT, resulting from impaired insulin action and glucose transport [42, 43]. This state favorizes hyperinsulinemia and the installation of insulin resistance. According to Rondinone et al. [44], insulin-stimulated AKT phosphorylation was impaired in the skeletal muscle of insulin-resistant Goto-Kakizaki rats and in muscle biopsies from type 2 diabetic patients [45]. Defects in GLUT4 translocation and expression were associated with the defective AKT phosphorylation [46].

In the present study,* Brassica rapa* treatments decrease the level of IRS1 p Ser and activate the AKT pathway, it could be associated with kaempferol which induced the activation of PI3 K and AKT in MC3T3-E1 cells [47].

Our results reveal that glucotoxicity increases inflammatory states in adipose tissue and VSMCs, marked by an increase of NO, MCP1, TNF*α*, and NF-*κ*B levels compared to the controls. Hyperglycemia induced on T cells increased synthesis of proinflammatory cytokines, which are responsible for the increase of NO by activation of the inducible nitric oxide synthase (iNOS) [34].

Glucotoxicity and insulin resistance activate the NF-*κ*B transcription factor and play a role in the induction of proinflammatory gene expression and the promotion of the inflammatory process in the vascular tissue. The initial event of inflammation in vascular tissue starts by the recruitment of monocytes in the lesions where MCP-1 plays a key role [48]. NF-*κ*B coordinates the induction of a wide range of genes encoding proinflammatory cytokines (interleukins 1, 2, and 6 and TNF*α*), chemokines (IL-8 and MCP1), adhesion molecules, acute-phase proteins, immune receptors, growth factors, and inducible enzymes such as vascular endothelial growth factor, cyclooxygenase-2 (COX-2), matrix metalloproteinases, iNOS, all molecules involved in inflammation other than in angiogenesis, cell proliferation, adhesion, migration, and invasion [49].

*Brassica* corrects the inflammatory states. This finding is in consistence with a previous study showing an improvement in the inflammatory states with the presence of polyphenol [50]. Quercetin, kaempferol, epigallocatechin gallate, and curcumin reduce the inflammatory markers (NO, MCP-1, TNF*α*, and NF-*κ*B) in diabetic experimental models [50, 51].

Quercetin has been demonstrated to inhibit NO production in LPS/cytokine-treated macrophages or macrophages-like cells by regulating iNOS protein expression and mRNA transcription [52, 53]. Recent data suggested that dietary polyphenols could exercise as modifiers of transduction signal pathways to elicit their beneficial effects [54]. Quercetin affects the upstream signaling of NF-*κ*B pathway by inhibiting upregulation members of the IKK complex, and these effects on the IKK cascade would in turn contribute to inhibition of NF-*κ*B activation [55]. In addition, the effect of quercetin on NF-*κ*B may be mediated through decreasing the phosphorylation state of IkB*α* and IkB*β*, which provided a direct mechanism, by which quercetin can inhibit the activity of NF-*κ*B, therefore decreasing endogenous expression of TNF*α* [55].

### 4.4. Glucotoxicity and Oxidative Stress Status 

The evaluation of antioxidant activity in blood (sera and erythrocytes) and VSMCs shows a decrease in catalase and SOD activity. The activities of SOD and catalase are low in diabetes mellitus. Excessive ROS generation can also be a causative factor for alteration in antioxidant enzymes expression and activity [56]. The AEBr increases the antioxidant enzymes activities in diabetic sand rats and in VSMCs submitted to glucotoxicity and this is by scavenging the toxic free radical, which is responsible for cells damage* in vivo* and* in vitro.*

The assessment of lipid peroxidation TBARs, in blood (sera and erythrocytes) and in VSMCs, significantly increased. According to the works of Hayek et al. [57] which observed that D-Glucose induces oxidative stress in macrophage by activating NADPH oxidase, thus increasing the production of superoxide ion which increases lipid peroxidation, including LDL. Hyperglycemia increases the genesis of ROS via activation of the polyol pathway and glycation, leading to lipid peroxidation [57, 58]. Lipid peroxidation mediated by ROS mainly targets the polyunsaturated fatty acids located at the cell membrane fluidity, modifying and altering membrane permeability; it affects the functions of proteins' membrane by the inactivation of receptors and activation of some proteases leading to cell damage [59]. The treatment with AEBr reduces the TBARs levels, in accordance with many studies in metabolic syndrome and diabetic rats treated with* Brassica* [18, 60]. Feillet-Coudray et al. [60] showed the preventive effect of polyphenols in rats fed a high-fat high sucrose diet.

Quercetin showed higher inhibitory effect on lipid peroxidation [61]. Kim et al. [62] report that ethanol extract of* Brassica rapa* attenuates oxidative stress. The reduction of oxidant markers* in vivo* and* in vitro* could be attributed to the presence of quercetin which is known to reduce TBARs level, scavenging free radicals, and chelating transitional metal ions and thus retarding oxidative degradation [63]. In addition, the antioxidant activity of* Brassica* is related to the presence of phenolic compound which can play an important role in neutralizing ROS via quenching singlet and triplet oxygen or decomposing peroxides [64].

The evaluation of proteins oxidation in VSMCs shows an increase in PC and AOPP levels in VSMCs submitted to glucotoxicity compared to the controls. Elevated protein carbonyl levels has been detected in diabetes [65, 66] and high plasma PC levels in diabetic children and adolescents without complications compared to control subjects, indicating that oxidative protein damage occurs at the onset of disease and tends to increase in the later stage. Furthermore, decreased antioxidant defenses might increase the susceptibility of diabetic patients to oxidative injury [67].* Brassica* decrease the levels of PC and AOPP by scavenging the toxic free radical. Polyphenols supplementation prevented, at least partially, the oxidation of lipid and protein induced in liver and heart of diabetic patients by probably limiting oxidative damage following acting directly on ROS, by acceptance of an electron by the phenolic groups or by stimulating endogenous defense systems [68].

Our results show a decrease of VSMCs viability submitted to glucotoxicity accompanied with an increase of cytochrome c and signs of apoptosis probably in relation to inhibition of AKT. We note therefore an increase of ERK1/2 which is related to phenotypic modification and hypertrophy. The treatment with* Brassica* increases cells viability, decreases the ERK1/2 and cytochrome levels, and prevents cell death by apoptosis. Our results are similar to Ye et al. [69] and Kapoor and Kakkar [70] and Piao et al. [71] who showed the beneficial effect of polyphenols contained, respectively, in the thea, morin, and brassica, in cultured cell under glucotoxicity conditions. Our results about ERK1/2 do not accord with the study of Heo et al. [72], saying that the proliferative effect of D-Glucose is related to the activation of MAPKinase via the pathway ERK1/2, which regulates the activity of Erg-1 and which plays a role in proliferation and dedifferentiation of vascular cells [73]. The elevation of ERK1/2 levels can implicate a phenotypic change of SMC into hypertrophied phenotype [74]. PI3K/AKT has been shown to play a major role in the prevention of apoptosis, and ERK1 is well-known, taking part in a transduction cascade signal in response to extracellular stimuli, and plays an important role in cell proliferation and cells growth and death [75, 76].

NF-*κ*B is extensively found in all kinds of cells. It has been shown to regulate the expression of numerous genes that play important roles in cellular stress responses, cells growth, survival, and apoptosis [77]. After prolonging exposure time of islet cells to high glucose, NF-*κ*B expression and cytochrome c release were significantly increased. These findings suggested that islets cells, exposed to glucotoxicity, may contribute on an induction of apoptosis involving cytochrome c release from mitochondria to cytosol, then pancreatic B-cells undergoing apoptosis [78]. Piro et al. [79] found that apoptosis of pancreatic islet cells was observed under high glucose and it was associated with an increase in the cytochrome c release and caspase-3 activation. Polyphenols are effective antioxidants, and have beneficial effects in metabolic disorders, by reducing cardiovascular complications associated with diabetes and metabolic syndrome [58].

## 5. Conclusion

In summary, we provide the evidence that* Brassica rapa* var.* rapifera* can reduce oxidative stress and inflammation in diabetic* Psammomys obesus*. It reduces hyperglycemia and hyperlipidemia and regulates the hypoinsulinemia in diabetic sand rats.* Brassica* induces changes with better cells survival and function* in vivo* (blood, heart, adipose tissue, and liver). In VSMCs of* Psammomys obesus in vitro, Brassica* reduces oxidized lipids and proteins gendered by the ROS and inflammatory markers by the modulation of IRS1-p-serine which is responsible of reducing TNF*α* liberation and inhibiting the liberation of MCP1, NF-*κ*B, and Cyt c. The AEBr enhance the activity of catalase and SOD. It can also activate the signaling pathways impaired in diabetes (AKT) and modulate the activity of ERK1/2. These results suggest that the naturally occurring aqueous extract of* Brassica rapa* may have an important implications for the prevention of type 2 diabetes mellitus and its complications and can be used as nutritional complement.

## Figures and Tables

**Table 1:** Total phenolic and flavonoids contentsand antioxidant activity of *Brassica rapa *var. *rapifera*.

Extract/standards	Total phenolic content (*µ*g GAE/mg)	Total flavonoids (*µ*g QE/mg)	DPPH (IC_50_)
Aqueous extract	9.41 ± 0.18	1.01 ± 0.09	2100 ± 13
BHA	n.a.	n.a.	21.28 ± 0.12
BHT	n.a.	n.a.	12.76 ± 0.08

Each value is expressed as means ± standard deviations for triplicate experiments. n.a.: not applied.

Quercetin (Q), quercetin equivalents (QE), gallic acid (GA), gallic acid equivalents (GAE), butylhydroxyanisole (BHA), and butylhydroxytoluene (BHT).

**(a):** 

Parameters	Control	HGD	Control + AEBr	HGD + AEBr
Glycemia (g L^−1^)	0.65 ± 0.055	1.63 ± 0.08^(*∗∗∗∗*)^	0.61 ± 0.045	1.27 ± 0.16^(*∗∗∗∗*,++++)^
TG (g L^−1^)	0.38 ± 0.08	3.11 ± 1.69^(*∗∗∗∗*)^	0.39 ± 0.09	1.09 ± 0.37^(*∗∗∗∗*,++++)^
Cholesterol (g L^−1^)	0.90 ± 0.06	6.65 ± 2.98^(*∗∗∗∗*)^	0.78 ± 0.12	2.08 ± 0.68^(*∗∗∗∗*,++++)^
HDL-cholesterol (mmol L^−1^)	1.89 ± 0.21	1.49 ± 0.15^(*∗∗∗*)^	1.99 ± 0.17	3.86 ± 0.39^(*∗∗∗∗*,++++)^
LDL- cholesterol (mmol L^−1^)	0.54 ± 0.26	25.82 ± 15.48^(*∗∗∗∗*)^	0.51 ± 0.18	0.42 ± 0.015^(++++)^
CPK (U L^−1^)	4 ± 1.00	436 ± 34.02^(*∗∗∗∗*)^	3 ± 0.82	42.50 ± 2.12^(*∗∗∗∗*,++++)^
LDH (U L^−1^)	4 ± 2.82	82 ± 56.66^(*∗∗∗∗*)^	3 ± 1.21	9.00 ± 6.24^(++++)^
Creatine (*µ*mol L^−1^)	28 ± 1.41	80 ± 49.98^(*∗∗∗∗*)^	26 ± 0.50	7.50 ± 0.71^(*∗∗∗∗*,++++)^

Data are expressed as mean ± standard deviation (SD); (*n* = 6). *Po*: *Psammomys obesus*, HGD: hyperglucidic diet, and HGD + AEBr: hyperglucidic diet treated with aqueous extract of *Brassica rapa*. *p* > 0.5 denotes a statistical difference; *p* < 0.5 statistically different.

*p* > 0.5 (*Po* control + AEBr versus *Po* control); ^*∗∗∗*^ *p* < 0.001 (*Po* HGD versus *Po* control), ^*∗∗∗∗*^ *p* < 0.0001 (*Po* HGD versus *Po* control), ^*∗∗∗∗*^ *p* < 0.0001 (*Po* HGD + AEBr versus *Po* control + AEBr); ^++++^ *p* < 0.0001 (*Po* HGD + AEBr versus *Po* HGD).

**(b):** 

Insulinemia (UI)	Control	Control + AEBr	HGD	HGD + AEBr
T0	25.2 ± 5.58	/	24.56 ± 6.20	/
T6	24.61 ± 6.20	/	358.66 ± 43.66^(*∗∗∗∗*)^	/
T final	36.2 ± 1.55	30.2 ± 1.00	10.36 ± 0.68^(*∗∗∗∗*)^	35.95 ± 0.85^(*∗*,++++)^

Data are expressed as mean ± standard deviation (SD); (*n* = 6). *Po*: *Psammomys obesus*, HGD: hyperglucidic diet, and HGD + AEBr: hyperglucidic diet treated with aqueous extract of *Brassica rapa*. *p* > 0.5 denotes a statistical difference; *p* < 0.5 statistically different.

*p* > 0.5 (*Po* control + AEBr versus *Po* control); ^*∗∗∗∗*^ *p* < 0.0001(*Po* HGD versus *Po* control); ^*∗*^ *p* < 0.01 (*Po* HGD + AEBr versus *Po* control + AEBr); ^++++^ *p* < 0.0001 (*Po* HGD + AEBr versus *Po* HGD).

**Table 3:** Changes of redox states in blood (sera and erythrocytes) in *Psammomys obesus* submitted to hyperglucidic diet (30% sucrose for 9 months). Effect of aqueous extract of *Brassica rapa *(AEBr) at 100 mg/kg for 20 consecutive days.

Redox states	Blood	Control	HGD	Control + AEBr	HGD + AEBr
Catalase (UI min^−1^ mg^−1^ of protein)	Sera	0.86 ± 0.24	0.23 ± 0.14^(*∗∗∗∗*)^	0.96 ± 0.34	1.22 ± 0.05^(++++)^
	Erythrocytes	1.3 ± 0.13	0.89 ± 0.1^(*∗∗∗∗*)^	1.33 ± 0.03	21.78 ± 2.55^(*∗∗∗∗*,++++)^

TBARs (*µ*M)	Sera	36.4 ± 2.65	46.7 ± 2.01^(*∗∗∗∗*)^	35.9 ± 1.65	39.5 ± 1.7^(*∗∗*,++++)^
	Erythrocytes	43.5 ± 0.7	53.8 ± 1.6^(*∗∗∗∗*)^	41.5 ± 0.17	43.8 ± 0.57^(++++)^

Data are expressed as mean ± standard deviation (S.D); (*n* = 6). *Po*: *Psammomys obesus*, HGD: hyperglucidic diet, and HGD + AEBr: hyperglucidic diet treated with aqueous extract of *Brassica rapa*. *p* > 0.5 denotes a statistical difference; *p* < 0.5 statistically different.

*p* > 0.5 (*Po* control + AEBr versus *Po* control); ^*∗∗*^ *p* < 0.01 (*Po* HGD + AEBr versus *Po* control + AEBr); ^*∗∗∗∗*^ *p* < 0.0001 (*Po* HGD versus *Po* control); ^*∗∗∗∗*^ *p* < 0.0001 (*Po* HGD + AEBr versus *Po* control + AEBr); ^++++^ *p* < 0.0001 (*Po* HGD + AEBr versus *Po* HGD).

**Table 4:** Changes of inflammatory markers in adipose tissue in* Psammomys obesus* submitted to hyperglucidic diet (30% sucrose for 9 months). Effect of aqueous extract of *Brassica rapa *(AEBr) at 100 mg/kg for 20 consecutive days.

Inflammatory markers	Control	HGD	Control + AEBr	HGD + AEBr
NF-*κ*B P35 (pg/100 mg of organs)	334 ± 13	587 ± 33^(*∗∗∗∗*)^	323 ± 3	408 ± 21^(++)^
TNFalpha (pg/100 mg of organs)	34.98 ± 1.8	134,2 ± 10^(*∗∗∗∗*)^	31.38 ± 13.1	46.6 ± 13.8^(++++)^
MCP1 (pg/100 mg of organs)	98.47 ± 6	274 ± 41^(*∗∗∗∗*)^	88.47 ± 5	102 ± 12^(*∗*,++++)^

Data are expressed as mean ± standard deviation (SD); (*n* = 6). *Po*: *Psammomys obesus*, HGD: hyperglucidic diet, and HGD + AEBr: hyperglucidic diet treated with aqueous extract of *Brassica rapa*. *p* > 0.5 denotes a statistical difference; *p* < 0.5 statistically different.

*p* > 0.5 (*Po* control + AEBr versus *Po* control); ^*∗*^ *p* < 0.5 (*Po* HGD versus *Po* control); ^*∗∗∗∗*^ *p* < 0.0001 (*Po* HGD versus *Po* control); ^++^ *p* < 0.001 (*Po* HGD + AEBr versus *Po* HGD); ^++++^ *p* < 0.0001 (*Po* HGD + AEBr versus *Po* HGD).

**Table 5:** Evaluation of inflammatory markers in vascular smooth muscle's cells (VSMCs) of* Psammomys obesus* submitted to glucotoxicity (Glc 0.6% for 48 hours) and treated with aqueous extract of *Brassica rapa* (AEBr) at 2100 *μ*g/mL for 24 hours.

Inflammation markers	Control	Glc	Control + AEBr	Glc + AEBr
NO (pmol/10^6^ cells)	16.28 ± 1.31	30.73 ± 3.62^(*∗∗∗∗*)^	23.71 ± 1.55	11.51 ± 3.48^(*∗∗∗∗*,++++)^
TNF*α* (pg/10^6^ cells)	14.46 ± 0.95	172.85 ± 9.32^(*∗∗∗∗*)^	17.20 ± 0.86	119.31 ± 4.25^(*∗∗∗∗*,++++)^
MCP1 (pg/10^6^ cells)	30.85 ± 4.68	164.48 ± 2.34^(*∗∗∗∗*)^	49.27 ± 10.17	80 ± 8.56^(*∗∗∗∗*,++++)^
NF-*κ*B P35 (pg/10^6^ cells)	17.86 ± 1.10	72.69 ± 2.72^(*∗∗∗∗*)^	16.58 ± 1.13	33.08 ± 0.59^(*∗∗∗∗*,++++)^

Data are expressed as mean ± standard deviation (SD); (*n* = 6). *Po*: *Psammomys obesus* and GLC: D-Glucose. *p* > 0.5 denotes a statistical difference; *p* < 0.5 statistically different.

*p* > 0.5 (*Po* VSMCs control + AEBr versus *Po* VSMCs control); ^*∗∗∗∗*^ *p* < 0.0001 (*Po* VSMCs Glc versus *Po* VSMCs control); ^*∗∗∗∗*^ *p* < 0.0001 (*Po* VSMCs Glc + AEBr versus *Po* control + AEBr); ^++++^ *p* < 0.0001 (*Po* VSMCs Glc + AEBr versus *Po* VSMCs Glc).

**Table 6:** Evaluation of redox states in vascular smooth muscle's cells (VSMCs) of* Psammomys obesus* submitted to glucotoxicity (Glc 0.6% for 48 hours) and treated with aqueous extract of *Brassica rapa* (AEBr) at 2100 *μ*g/mL for 24 hours.

Redox states	Control	Glc	Control + AEBr	Glc + AEBr
Catalase (UI/min^−1^ mg^−1^ protein ×10^6^ cells)	0.102 ± 0.005	0.031 ± 0.012^(*∗∗∗∗*)^	0.067 ± 0.029	0.158 ± 0.008^(*∗∗∗∗*,++++)^
SOD (UI/mg^−1^ protein ×10^6^ cells)	1.38 ± 0	0.68 ± 0.01^(*∗∗∗∗*)^	1.14 ± 0	2.07 ± 0.16^(*∗∗∗∗*,++++)^

TBARs CIC (*µ*mol/10^6^ cells)	1.08 ± 0.05	1.71 ± 0.15^(*∗∗∗∗*)^	0.49 ± 0.13	0.39 ± 0.05^(++++)^
TBARs CEC (*µ*mol/10^6^ cells)	0.85 ± 0.01	2.32 ± 0.02^(*∗∗∗∗*)^	1.13 ± 0.034	0.91 ± 0.02^(*∗∗∗*,++++)^
protein carbonyls (pmol/10^6^ cells)	0.77 ± 0.21	10.52 ± 0.23^(*∗∗∗∗*)^	0.76 ± 0.11	2.48 ± 0.12^(*∗∗∗∗*,++++)^
AOPP (pmol/10^6^ cells)	216.09 ± 10.8	349.67 ± 37.05^(*∗∗∗∗*)^	209.65 ± 35.6	93.72 ± 9.11^(*∗∗∗∗*,++++)^

Data are expressed as mean ± standard deviation (SD); (*n* = 6). *Po*: *Psammomys obesus*, Glc: D-Glucose, SOD: superoxide dismutase, and AOPP: advanced protein oxidation products. *p* > 0.5 denotes a statistical difference; *p* < 0.5 statistically different.

*p* > 0.5 (*Po* control + AEBr versus *Po* control); ^*∗∗∗*^ *p* < 0.001 (*Po* VSMCs Glc + AEBr versus *Po* control + AEBr); ^*∗∗∗∗*^ *p* < 0.0001 (*Po* VSMCs Glc versus *Po* VSMCs control); ^*∗∗∗∗*^ *p* < 0.0001 (*Po* VSMCs Glc + AEBr versus *Po* control + AEBr); ^++++^ *p* < 0.0001 (*Po* VSMCs Glc + AEBr versus *Po* VSMCs Glc).

## Abbreviations

AEBr: Aqueous extract of* Brassica rapa*
AOPP: Advanced protein oxidation products
BHA: Butylhydroxyanisole
BHT: Butylhydroxytoluene
COX-2: Cyclooxygenase-2
CPK: Creatine phosphokinase
Cyt c: Cytochrome c
DM: Diabetes mellitus
DMEM: Dulbecco's modified Eagle's medium
DNPH: Dinitrophenylhydrazine
DPPH: 2,2-Diphenyl-1-picrylhydrazyl
E: Equivalents
FCS: Fetal calf serum
GA: Gallic acid
Glc: D-Glucose
HGD: Hyperglucidic diet group
HGD + AEBr: Hyperglucidic diet treated with aqueous extract of* Brassica rapa*
iNOS: Inducible nitric oxide synthase
LDH: Lactic dehydrogenase
MDA: Malondialdehyde
MGG: May Grunwald-Giemsa
NBT: Nitroblue tetrazolium
NO: Nitrogen monoxide
PC: Protein carbonyls
*Po*: * Psammomys obesus*
Q: Quercetin
ROS: Reactive oxygen species
SD: Standard deviation
SOD: Superoxyde dismutase
TBA: Thiobarbituric acid
TBARs: Thiobarbituric acid reactive substances
VSMCs: Vascular smooth muscle cells.

## References

[1] IDF Diabetes Atlas (2011). *International Diabetes Federation*.

[2] Abdollahi M., Ranjbar A., Shadnia S., Nikfar S., Rezaie A. (2004). Pesticides and oxidative stress: a review. *Medical Science Monitor*.

[3] Rahimi R., Nikfar S., Larijani B., Abdollahi M. (2005). A review on the role of antioxidants in the management of diabetes and its complications. *Biomedicine & Pharmacotherapy*.

[4] Schaffer S. W., Jong C. J., Mozaffari M. (2012). Role of oxidative stress in diabetes-mediated vascular dysfunction: unifying hypothesis of diabetes revisited. *Vascular Pharmacology*.

[5] Bahadoran Z., Mirmiran P., Azizi F. (2013). Dietary polyphenols as potential nutraceuticals in management of diabetes: a review. *Journal of Diabetes and Metabolic Disorders*.

[6] Brunner Y., Schvartz D., Priego-Capote F., Couté Y., Sanchez J.-C. (2009). Glucotoxicity and pancreatic proteomics. *Journal of Proteomics*.

[7] Pandolfi A., De Filippis E. A. (2007). Chronic hyperglicemia and nitric oxide bioavailability play a pivotal role in pro-atherogenic vascular modifications. *Genes and Nutrition*.

[8] Andreea S. I., Marieta C., Anca D. (2008). AGEs and glucose levels modulate type I and III procollagen mRNA synthesis in dermal fibroblasts cells culture. *Experimental Diabetes Research*.

[9] Xu Y., Nie L., Yin Y.-G. (2012). Resveratrol protects against hyperglycemia-induced oxidative damage to mitochondria by activating SIRT1 in rat mesangial cells. *Toxicology and Applied Pharmacology*.

[10] Romani A., Vignolini P., Isolani L., Ieri F., Heimler D. (2006). HPLC-DAD/MS characterization of flavonoids and hydroxycinnamic derivatives in turnip tops (*Brassica rapa* L. Subsp. *sylvestris* L.). *Journal of Agricultural and Food Chemistry*.

[11] Schonhof I., Krumbein A., Brückner B. (2004). Genotypic effects on glucosinolates and sensory properties of broccoli and cauliflower. *Nahrung/Food*.

[12] Jung U. J., Baek N.-I., Chung H.-G. (2008). Effects of the ethanol extract of the roots of *Brassica rapa* on glucose and lipid metabolism in C57BL/KsJ-db/db mice. *Clinical Nutrition*.

[13] Daryoush M., Bahram A. T., Yousef D., Mehrdad N. (2011). *Brassica rapa* L. extract alleviate early hepatic injury in alloxan-induced diabetic rats. *Journal of Medicinal Plant Research*.

[14] Abo-youssef A. M., Mohammed R. (2013). Effects of *Brassica rapa* on fructose-induced metabolic syndrome in rats: a comparative study. *International Journal of Pharmaceutical Sciences Review and Research*.

[15] Daly M., Daly S. (1973). On the feeding ecology of *Psammomys obesus* (Rodentia, Gerbillidae) in the Wadi Saoura, Algeria. *Mammalia*.

[16] Singleton V. L., Orthofer R., Lamuela-Raventós R. M. (1998). Analysis of total phenols and other oxidation substrates and antioxidants by means of folin-ciocalteu reagent. *Methods in Enzymology*.

[17] Djouahri A., Boudarene L., Saka B. (2014). In vitro synergistic/antagonistic antibacterial and anti-inflammatory effect of various extracts/essential oil from cones of Tetraclinis articulata (Vahl) Masters with antibiotic and anti-inflammatory agents. *Industrial Crops and Products*.

[18] Brand-Williams W., Cuvelier M. E., Berset C. (1995). Use of a free radical method to evaluate antioxidant activity. *LWT—Food Science and Technology*.

[19] Berdja S., Smail L., Othmani K. (2012). Impact of glucotoxicity induced *in vivo* and *in vitro* in *Psammomys obesus*. *Journal of Diabetes Mellitus*.

[20] Bourdillon M. C., Boissel J. P., Crouzet B. (1977). Proliferation of primary cultures from rat aortic media. Effects of hyperlipidemic serum. *Progress in Biochemical Pharmacology*.

[21] Aouichat Bouguerra S., Bourdillon M. C., Dahmani Y., Bekkhoucha F. (2001). Non insulin dependent diabetes in sand rat (*Psammomys obesus*) and production of collagen in cultured aortic smooth muscle cells. Influence of insulin. *International Journal of Experimental Diabetes Research*.

[22] Claiborne A. (1985). Catalase activity. *CRC Handbook of Methods for Oxygen Radical Research*.

[23] Sun Y., Oberley L. W., Li Y. (1988). A simple method for clinical assay of superoxide dismutase. *Clinical Chemistry*.

[24] Giannopolitis C. N., Ries S. K. (1977). Superoxide dismutases occurrence in higher plants. *Plant Physiology*.

[25] Heath R. L., Packer L. (1968). Photoperoxidation in isolated chloroplasts. I. Kinetics and stoichiometry of fatty acid peroxidation. *Archives of Biochemistry and Biophysics*.

[26] Grand F., Guitton J., Goudable J. (2001). Optimisation des parametres du dosage des nitrites et nitrates seriques par la technique de Griess. *Annales de Biologie Clinique*.

[27] Reznick A. Z., Packer L. (1994). Oxidative damage to proteins: spectrophotometric method for carbonyl assay. *Methods in Enzymology*.

[28] Witko-Sarsat V., Friedlander M., Capeillère-Blandin C. (1996). Advanced oxidation protein products as a novel marker of oxidative stress in uremia. *Kidney International*.

[29] Çakatay U. (2005). Protein oxidation parameters in type 2 diabetic patients with good and poor glycaemic control. *Diabetes & Metabolism*.

[30] Decker E. A. (1997). Phenolics: prooxidants or antioxidants?. *Nutrition Reviews*.

[31] Al-Mamary M., Al-Meeri A., Al-Habori M. (2002). Antioxidant activities and total phenolics of different types of honey. *Nutrition Research*.

[32] Marquie G., Duhault J., Jacotot B. (1984). Diabetes mellitus in sand rats (*Psammomys obesus*). Metabolic pattern during development of the diabetic syndrome. *Diabetes*.

[33] Shafrir E. (2001). Albert Renold memorial lecture: molecular background of nutritionally induced insulin resistance leading to type 2 diabetes—from animal models to humans. *The International Journal of Experimental Diabetes Research*.

[34] Stentz F. B., Kitabchi A. E. (2005). Hyperglycemia-induced activation of human T-lymphocytes with de novo emergence of insulin receptors and generation of reactive oxygen species. *Biochemical and Biophysical Research Communications*.

[35] Navab M., Hama S. Y., Ready S. T. (2002). Oxidized lipids as mediators of coronary heart disease. *Current Opinion in Lipidology*.

[36] Brewer H. B. (2004). High density lipoprotein: a new potential therapeutic target for the prevention of cardio-vascular disease. *Arteriosclerosis, Thrombosis, and Vascular Biology*.

[37] Anand P., Murali K. Y., Tandon V., Chandra R., Murthy P. S. (2007). Preliminary studies on antihyperglycemic effect of aqueous extract of *Brassica nigra* (L.) Koch in streptozotocin induced diabetic rats. *Indian Journal of Experimental Biology*.

[38] Bhowmik A., Khan L. A., Akhter M., Rokeya B. (2009). Studies on the antidiabetic effects of Mangifera indica stem-barks and leaves on nondiabetic, type 1 and type 2 diabetic model rats. *Bangladesh Journal of Pharmacology*.

[39] Nyunaï N., Njikam N., Abdennebi E. H., Mbafor J. T., Lamnaouer D. (2009). Hypoglycaemic and antihyperglycaemic activity of *Ageratum conyzoides* L. in rats. *African Journal of Traditional, Complementary and Alternative Medicines*.

[40] Baudouy P. Y., Beaufils P. (1998). Diagnostic de l'infarctus du myocarde aigu. *Encyclopédie Médico—Chirurgicale Cardiologie*.

[41] Kataya H. A. H., Hamza A. A. (2008). Red cabbage (*Brassica oleracea*) ameliorates diabetic nephropathy in rats. *Evidence- Based Complementary and Alternative Medicine*.

[42] Kim J.-A., Montagnani M., Kwang K. K., Quon M. J. (2006). Reciprocal relationships between insulin resistance and endothelial dysfunction: molecular and pathophysiological mechanisms. *Circulation*.

[43] Evans J. L., Goldfine I. D., Maddux B. A., Grodsky G. M. (2002). Oxidative stress and stress-activated signaling pathways: a unifying hypothesis of type 2 diabetes. *Endocrine Reviews*.

[44] Rondinone C. M., Carvalho E., Wesslau C., Smith U. P. (1999). Impaired glucose transport and protein kinase B activation by insulin, but not okadaic acid, in adipocytes from subjects with Type II diabetes mellitus. *Diabetologia*.

[45] Krook A., Kawano Y., Song X. M. (1997). Improved glucose tolerance restores insulin-stimulated Akt kinase activity and glucose transport in skeletal muscle from diabetic Goto-Kakizaki rats. *Diabetes*.

[46] Carvalho E., Rondinone C., Smith U. (2000). Insulin resistance in fat cells from obese zucker rats—evidence for an impaired activation and translocation of protein kinase B and glucose transporter 4. *Molecular and Cellular Biochemistry*.

[47] Choi E. M. (2011). Kaempferol protects MC3T3-E1 cells through antioxidant effect and regulation of mitochondrial function. *Food and Chemical Toxicology*.

[48] Dagre A. G., Lekakis J. P., Protogerou A. D. (2007). Abnormal endothelial function in female patients with hypothyroidism and borderline thyroid function. *The International Journal of Cardiology*.

[49] Santangelo C., Varì R., Scazzocchio B., Di Benedetto R., Filesi C., Masella R. (2007). Polyphenols, intracellular signalling and inflammation. *Annali dell'Istituto Superiore di Sanita*.

[50] Mahmoud M. F., Hassan N. A., El Bassossy H. M., Fahmy A. (2013). Quercetin protects against diabetes-induced exaggerated vasoconstriction in rats: effects on low grade inflammation. *PLoS ONE*.

[51] Margina D., Gradinaru D., Manda G., Neagoe I., Ilie M. (2013). Membranar effects exerted in vitro by polyphenols—quercetin, epigallocatechin gallate and curcumin—on HUVEC and Jurkat cells, relevant for diabetes mellitus. *Food and Chemical Toxicology*.

[52] Wadsworth T. L., Koop D. R. (1999). Effects of the wine polyphenolics quercetin and resveratrol on pro-inflammatory cytokine expression in RAW 264.7 macrophages. *Biochemical Pharmacology*.

[53] Chen Y. C., Shen S. C., Lee W. R., Hou W. C., Yang L. L., Lee T. J. F. (2001). Inhibition of nitric oxide synthase inhibitors and lipopolysaccharide induced inducible NOS and cyclooxygenase-2 gene expressions by rutin, quercetin, and quercetin pentaacetate in RAW 264.7 macrophages. *Journal of Cellular Biochemistry*.

[54] Rahman I., Biswas S. K., Kirkham P. A. (2006). Regulation of inflammation and redox signaling by dietary polyphenols. *Biochemical Pharmacology*.

[55] Nam N.-H. (2006). Naturally occurring NF-*κ*B inhibitors. *Mini-Reviews in Medicinal Chemistry*.

[56] Vucic M., Gavella M., Bozikov V., Ashcroft S. J. H., Rocic B. (1997). Superoxide dismutase activity in lymphocytes and polymorphonuclear cells of diabetic patients. *European Journal of Clinical Chemistry and Clinical Biochemistry*.

[57] Hayek T., Kaplan M., Kerry R., Aviram M. (2007). Macrophage NADPH oxidase activation, impaired cholesterol fluxes, and increased cholesterol biosynthesis in diabetic mice: a stimulatory role for D-glucose. *Atherosclerosis*.

[58] Whiteside C. I., Dlugosz J. A. (2002). Mesangial cell protein kinase C isoenzyme activation in the diabetic milieu. *The American Journal Physiology—Renal Physiology*.

[59] Michel F., Bonnefont-Rousselot D., Mas E., Drai J., Thérond P. (2008). Biomarqueurs de la peroxydation lipidiques: aspects analytiques. *Annales de Biologie Clinique*.

[60] Feillet-Coudray C., Sutra T., Fouret G. (2009). Oxidative stress in rats fed a high-fat high-sucrose diet and preventive effect of polyphenols: involvement of mitochondrial and NAD(P)H oxidase systems. *Free Radical Biology & Medicine*.

[61] Rasal V., Shetty B., Sinnathambi A., Yeshmania S., Ashok P. (2005). Antihyperglycaemic and antioxidant activity of *Brassica oleracea* in streptozotocin diabetic rats. *The Internet Journal of Pharmacology*.

[62] Kim Y.-H., Kim Y.-W., Oh Y.-J. (2006). Protective effect of the ethanol extract of the roots of *Brassica rapa* on cisplatin-induced nephrotoxicity in LLC-PK1 cells and rats. *Biological and Pharmaceutical Bulletin*.

[63] Mullen W., Marks S. C., Crozier A. (2007). Evaluation of phenolic compounds in commercial fruit juices and fruit drinks. *Journal of Agricultural and Food Chemistry*.

[64] Fresco P., Borges F., Marques M. P. M., Diniz C. (2010). The anticancer properties of dietary polyphenols and its relation with apoptosis. *Current Pharmaceutical Design*.

[65] Telci A., Çakatay U., Salman S., Satman I., Sivas A. (2000). Oxidative protein damage in early stage Type 1 diabetic patients. *Diabetes Research and Clinical Practice*.

[66] Telci A., Cakatay U., Kayali R. (2000). Oxidative protein damage in plasma of type 2 diabetic patients. *Hormone and Metabolic Research*.

[67] Dominguez C., Ruiz E., Gussinye M., Carrascosa A. (1998). Oxidative stress at onset and in early stages of type 1 diabetes in children and adolescents. *Diabetes Care*.

[68] Scalbert A., Manach C., Morand C., Rémésy C., Jiménez L. (2005). Dietary polyphenols and the prevention of diseases. *Critical Reviews in Food Science and Nutrition*.

[69] Ye P., Lin K., Li Z., Liu J., Yao K., Xu W. (2013). (−)-Epigallocatechin gallate regulates expression of apoptotic genes and protects cultured human lens epithelial cells under hyperglycemia. *Molekuliarnaia Biologiia*.

[70] Kapoor R., Kakkar P. (2012). Protective role of morin, a flavonoid, against high glucose induced oxidative stress mediated apoptosis in primary rat hepatocytes. *PLoS ONE*.

[71] Piao X. L., Kim H. Y., Yokozawa T., Lee Y. A., Piao X. S., Cho E. J. (2005). Protective effects of Broccoli (*Brassica oleracea*) and its active components against radical-induced oxidative damage. *Journal of Nutritional Science and Vitaminology*.

[72] Heo K.-S., Kim D.-U., Kim L. (2008). Activation of PKC*β*II and PKC*θ* is essential for LDL-induced cell proliferation of human aortic smooth muscle cells via Gi-mediated Erk1/2 activation and Egr-1 upregulation. *Biochemical and Biophysical Research Communications*.

[73] McCaffrey T. A., Fu C., Du B. (2000). High-level expression of Egr-1 and Egr-1-inducible genes in mouse and human atherosclerosis. *The Journal of Clinical Investigation*.

[74] Grimm D., Jabusch H. C., Kossmehl P. (2002). Experimental diabetes and left ventricular hypertrophy: effects of beta-receptor blockade. *Cardiovascular Pathology*.

[75] Martelli A. M., Faenza I., Billi A. M. (2006). Intranuclear 3′-phosphoinositide metabolism and Akt signaling: new mechanisms for tumorigenesis and protection against apoptosis?. *Cellular Signalling*.

[76] Karin M., Ben-Neriah Y. (2000). Phosphorylation meets ubiquitination: the control of NF-*κ*B activity. *Annual Review of Immunology*.

[77] Lu Z., Xu S. (2006). ERK1/2 MAP kinases in cell survival and apoptosis. *IUBMB Life*.

[78] Liang Y., Zhang M., Ning N. X., Yank Y., Feng L. (2009). Effects of high concentration glucose on the expression of NF-*κ*B, Bax and cytochrome C and apoptosis of islet cells in mice. *Journal of Huazhong University of Science and Technology. Medical Sciences*.

[79] Piro S., Anello M., Di Pietro C. (2002). Chronic exposure to free fatty acids or high glucose induces apoptosis in rat pancreatic islets: possible role of oxidative stress. *Metabolism: Clinical and Experimental*.

